# Supplementary material for: High diversity of fungal ecological groups in Andean–Patagonian Nothofagus forests
Source: PLoS One. 2023 Aug 24;18(8):e0290398. doi: 10.1371/journal.pone.0290398 (PMC10449153; doi:10.1371/journal.pone.0290398)
Supplement: S1 Fig — (PDF) [file pone.0290398.s007.pdf]

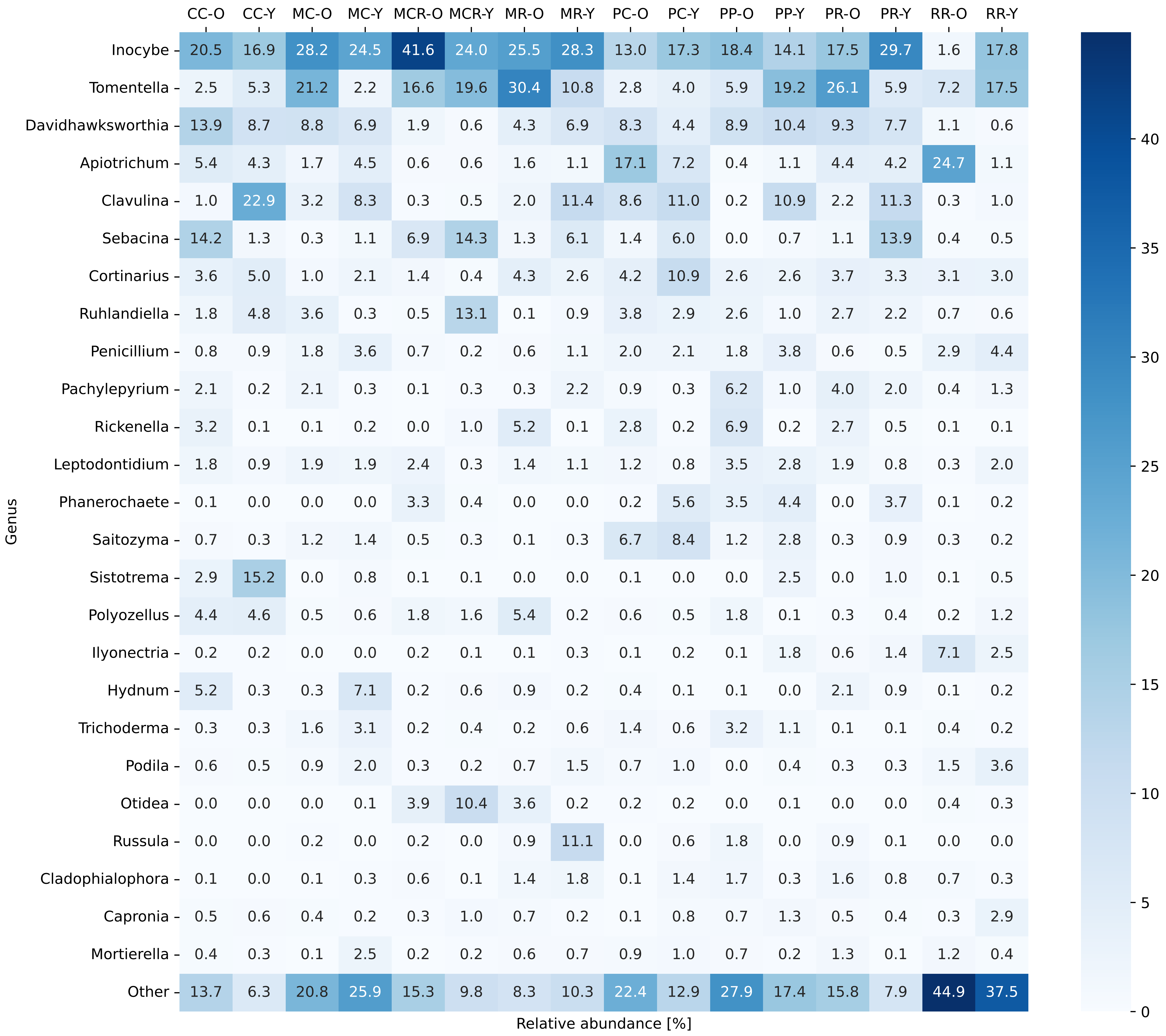
CC-O CC-Y MC-O MC-Y MCR-O MCR-Y MR-O MR-Y PC-O PC-Y PP-O PP-Y PR-O PR-Y RR-O RR-Y
Inocybe 20.5 16.9 28.2 24.5 41.6 24.0 25.5 28.3 13.0 17.3 18.4 14.1 17.5 29.7 1.6 17.8
Tomentella 2.5 5.3 21.2 2.2 16.6 19.6 30.4 10.8 2.8 4.0 5.9 19.2 26.1 5.9 7.2 17.5
Davidhawksworthia 13.9 8.7 8.8 6.9 1.9 0.6 4.3 6.9 8.3 4.4 8.9 10.4 9.3 7.7 1.1 0.6
Apiotrichum 5.4 4.3 1.7 4.5 0.6 0.6 1.6 1.1 17.1 7.2 0.4 1.1 4.4 4.2 24.7 1.1
Clavulina 1.0 22.9 3.2 8.3 0.3 0.5 2.0 11.4 8.6 11.0 0.2 10.9 2.2 11.3 0.3 1.0
Sebacina 14.2 1.3 0.3 1.1 6.9 14.3 1.3 6.1 1.4 6.0 0.0 0.7 1.1 13.9 0.4 0.5
Cortinarius 3.6 5.0 1.0 2.1 1.4 0.4 4.3 2.6 4.2 10.9 2.6 2.6 3.7 3.3 3.1 3.0
Ruhlandiella 1.8 4.8 3.6 0.3 0.5 13.1 0.1 0.9 3.8 2.9 2.6 1.0 2.7 2.2 0.7 0.6
Penicillium 0.8 0.9 1.8 3.6 0.7 0.2 0.6 1.1 2.0 2.1 1.8 3.8 0.6 0.5 2.9 4.4
Pachylepyrium 2.1 0.2 2.1 0.3 0.1 0.3 0.3 2.2 0.9 0.3 6.2 1.0 4.0 2.0 0.4 1.3
Rickenella 3.2 0.1 0.1 0.2 0.0 1.0 5.2 0.1 2.8 0.2 6.9 0.2 2.7 0.5 0.1 0.1
Leptodontidium 1.8 0.9 1.9 1.9 2.4 0.3 1.4 1.1 1.2 0.8 3.5 2.8 1.9 0.8 0.3 2.0
Phanerochaete 0.1 0.0 0.0 0.0 3.3 0.4 0.0 0.0 0.2 5.6 3.5 4.4 0.0 3.7 0.1 0.2
Saitozyma 0.7 0.3 1.2 1.4 0.5 0.3 0.1 0.3 6.7 8.4 1.2 2.8 0.3 0.9 0.3 0.2
Sistotrema 2.9 15.2 0.0 0.8 0.1 0.1 0.0 0.0 0.1 0.0 0.0 2.5 0.0 1.0 0.1 0.5
Polyozellus 4.4 4.6 0.5 0.6 1.8 1.6 5.4 0.2 0.6 0.5 1.8 0.1 0.3 0.4 0.2 1.2
Ilyonectria 0.2 0.2 0.0 0.0 0.2 0.1 0.1 0.3 0.1 0.2 0.1 1.8 0.6 1.4 7.1 2.5
Hydnum 5.2 0.3 0.3 7.1 0.2 0.6 0.9 0.2 0.4 0.1 0.1 0.0 2.1 0.9 0.1 0.2
Trichoderma 0.3 0.3 1.6 3.1 0.2 0.4 0.2 0.6 1.4 0.6 3.2 1.1 0.1 0.1 0.4 0.2
Podila 0.6 0.5 0.9 2.0 0.3 0.2 0.7 1.5 0.7 1.0 0.0 0.4 0.3 0.3 1.5 3.6
Otidea 0.0 0.0 0.0 0.1 3.9 10.4 3.6 0.2 0.2 0.2 0.0 0.1 0.0 0.0 0.4 0.3
Russula 0.0 0.0 0.2 0.0 0.2 0.0 0.9 11.1 0.0 0.6 1.8 0.0 0.9 0.1 0.0 0.0
Cladophialophora 0.1 0.0 0.1 0.3 0.6 0.1 1.4 1.8 0.1 1.4 1.7 0.3 1.6 0.8 0.7 0.3
Capronia 0.5 0.6 0.4 0.2 0.3 1.0 0.7 0.2 0.1 0.8 0.7 1.3 0.5 0.4 0.3 2.9
Mortierella 0.4 0.3 0.1 2.5 0.2 0.2 0.6 0.7 0.9 1.0 0.7 0.2 1.3 0.1 1.2 0.4
Other 13.7 6.3 20.8 25.9 15.3 9.8 8.3 10.3 22.4 12.9 27.9 17.4 15.8 7.9 44.9 37.5
Genus
Relative abundance [%]
0
5
10
15
20
25
30
35
40
