# Supplementary figures and images for: High diversity of fungal ecological groups in Andean–Patagonian Nothofagus forests
Source: PLoS One. 2023 Aug 24;18(8):e0290398. doi: 10.1371/journal.pone.0290398 (PMC10449153; doi:10.1371/journal.pone.0290398)

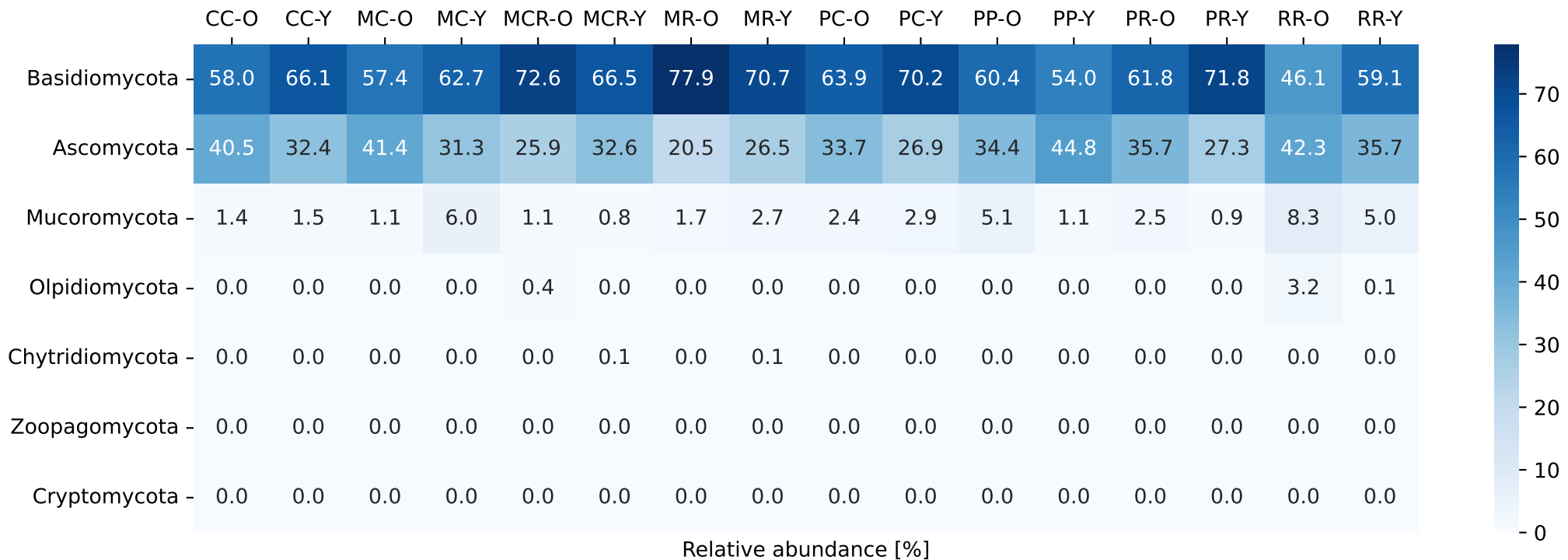

Supplement: S2 Fig — (PDF) [file pone.0290398.s008.pdf]

Diversity

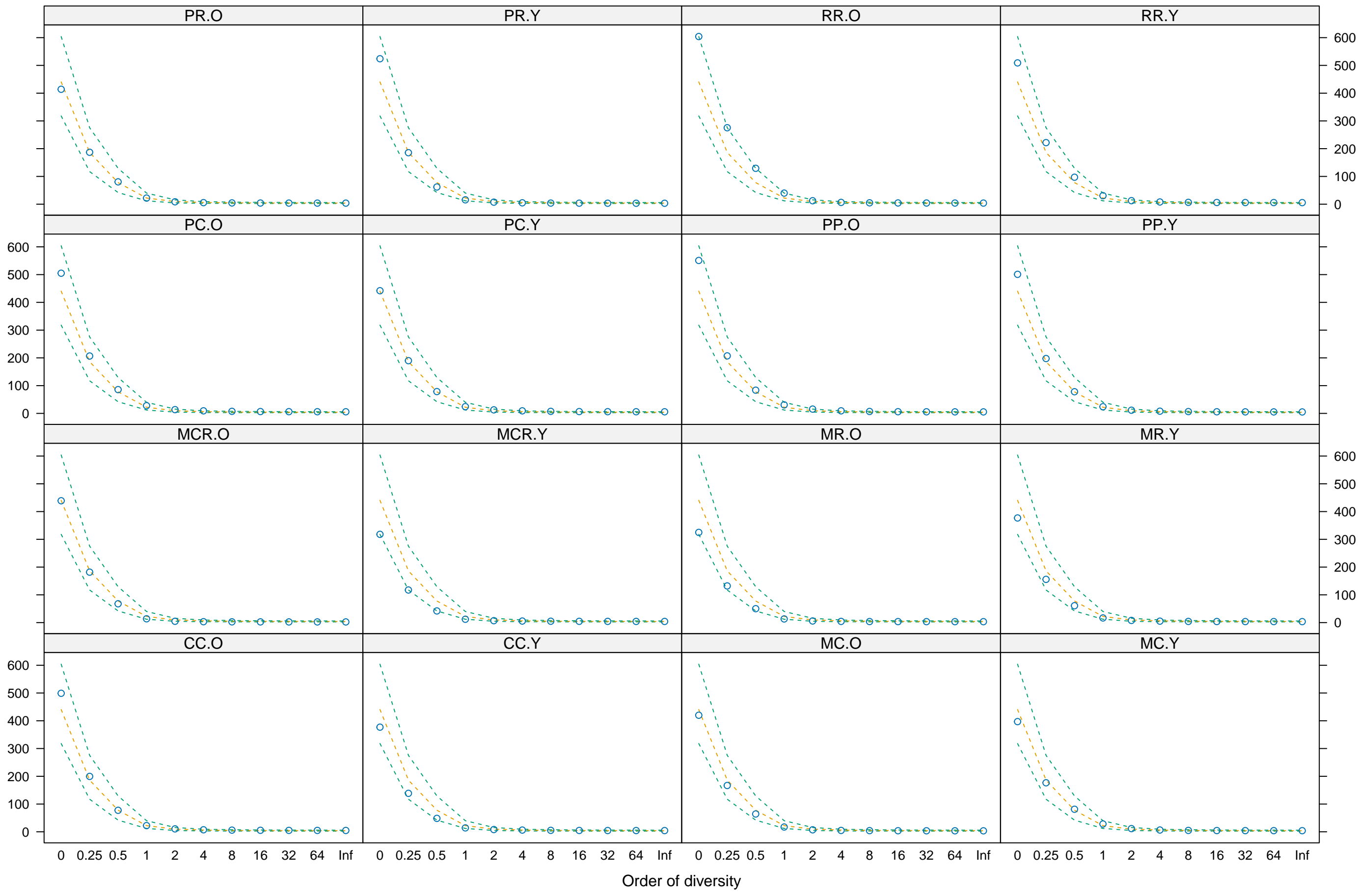

Order of diversity

Supplement: S3 Fig — (PDF) [file pone.0290398.s009.pdf]
