# Supplementary material for: High diversity of fungal ecological groups in Andean–Patagonian Nothofagus forests
Source: PLoS One. 2023 Aug 24;18(8):e0290398. doi: 10.1371/journal.pone.0290398 (PMC10449153; doi:10.1371/journal.pone.0290398)
Supplement: Attachment — Submitted filename: Text.pdf [file pone.0290398.s010.pdf]

Metabarcoding of root-associated ectomycorrhizal fungal communities in andean-patagonian *Nothofagus* forests

Max Emil Schön[1]*, Sarah Zuern[2], Milena Vera[2] and Sigisfredo Garnica[2]*

[1]Department of Biomolecular Mechanisms, Max Planck Institute for Medical Research, Heidelberg, Germany
[2]Instituto de Bioquímica y Microbiología, Facultad de Ciencias, Universidad Austral de Chile, Isla Teja, 5090000 Valdivia, Chile

Corresponding authors:

Sigisfredo Garnica*
Email: sigisfredo.garnica@uach.cl

Max Emil Schön*
Email: max-emil.schoen@mr.mpg.de

Abstract

Native andean-patagonian *Nothofagus* forests harbour a unique diversity of microorganisms with diverse ecological roles. Although ectomycorrhizal associations constitute an important fragment of the biota associated with these forests, the factors affecting such communities are largely unknown. We investigate the biodiversity, relative abundance, and composition of ectomycorrhizal fungal communities in relation to two host-tree species and age and soil properties in six monospecific and mixed evergreen-deciduous *Nothofagus* forests. We used the ITS2 region by sequencing 9.600 ECM root tips for the identification of fungi. A total of 1125 fungal taxa on the genus level distributed over 131 orders were identified. The phyla Ascomycota (34.5%) and Basidiomycota (62.1%) were most abundant, whereas Mucoromycota (3.1%), Chytridiomycota, Cryptomycota, Olpidiomycota, and Zoopagomycota occurred less frequently. The highest taxon diversity was found in mature trees, whereas young trees often exhibited lower diversity of associated fungi. The fungal taxa were grouped into 7 broad ecological categories of which saprotroph associations were most common, followed of pathotroph, pathotroph-saprotroph-symbiotroph, pathotroph-saprotroph, and symbiotroph. We did not detect major differences in relation to the number of taxa in each category between young and old *N. dombeyi* and *N. obliqua*. Overall, the scale of the metabarcoding approach allowed us to detect fungal taxa diversity that would not be possible to find through fruiting body surveys alone and that have never been observed in Nothofagus forests before. Our findings suggest an impact of the proximity between sites, similarity of soil conditions and anthropogenic use of the forests on the belowground fungal community's diversity and composition. Furthermore, there were differences between above- and belowground occurrences of the edible mushrooms *B. loyo* and *Ramaria* spp. However, future research including EMC tips found beneath fairy rings significantly could provide better correlation with the above-ground fruiting body occurrences.

Introduction

Fungi play pivotal ecological roles in forest ecosystems and some of them represent an important forest resource (cite). They may participate in the decomposition of wood and litter recycling of carbon, minerals and nutrients that ~~can be used by other organisms~~other organisms can use (cite). Alternatively, they can act as pathogens killing trees~~ and thereby altering the plant diversity or collaborate symbiotically with plants and promote~~, altering plant diversity or collaborating symbiotically with plants and promoting forest growth [1].

Research on fungi in forest ecosystems comes mostly from boreal and temperate forests, especially from North America and Europe (cite). In contrast, our knowledge of these microorganisms from other regions, such as the temperate forests of South America, is still very fragmentary. This applies both to their aboveground fruiting bodies and to their underground life, mostly studied by root tip sequencing (as reviewed by Barroetaveña et al. [2]). Recently, a high diversity of fungi, far beyond that detected from fruiting bodies, has been revealed to be associated with *Nothofagus* species [3–5]. Members of this genus, also known as the southern beeches, constitute the main part of forests in the southernmost parts of Chile and Argentina, but also in parts of Australia, New Zealand and New Guinea. Understanding the fungal partners of these trees with also help to elucidate factors impacting such less-well studied forest ecosystems.

An important functional group of wild mushrooms are those that establish obligatory symbiotic associations with roots of evergreen-deciduous *Nothofagus* species known as ectomycorrhizae (ECM). Nouhra et al. [3] found no significant differences between different Nothofagus species linked to diversity and composition of ECM fungal communities, but ~~there was an effect of altitude on~~altitude affected structure. Truong et al. [4] found similar functional fungal groups across elevations in *N. pumilio* forests in Chile and Argentina, and Almonacid-Muñoz et al. [5] found that microbial communities in ~~*Nothofagus* ~~N. *obliqua* are affected by surrounding forest and site characteristics.

In Chile, fungi growing in native forests are considered non-timber forest products [6]. Some wild mushroom species are also important as food, especially for indigenous Mapuche communities [7,8]. Despite their ecological importance and, for some of them, as a source of food [9], their occurrence as evaluated through the production of fruiting bodies, is apparently

decreasing due to anthropogenic pressures, especially the loss, alteration and/or fragmentation of their habitats [10]. Thus, research aimed at expanding knowledge about the biotic and abiotic factors that influence wild mushroom communities is vital for their management and conservation.

In this work, temperate rainforests were sampled mostly in the commune of Panguipulli, which is recognized by UNESCO as a Biosphere Reserve [11]; it is the commune with the largest area of native forest and Mapuche population in the Los Ríos region of Chile. The native forest covers more than 95% and *Nothofagus* in turn covers 66.7% of the communal surface. The two species *N. dombeyi* (evergreen) and *N. obliqua* (deciduous) are the dominant representatives [12]. ~~In this commune, a~~A~~round 42% of the population~~Around 42% of the population in this commune are members of the Mapuche people [13]. This aboriginal population still preserves many elements of their identity and culture, including knowledge of the natural environment [11,14]. In particular, ~~the gathering of wild mushrooms in these forests is a cultural practice that in recent years has disappeared more rapidly~~gathering wild mushrooms in these forests is a cultural practice that has disappeared more rapidly in recent years. This is due in part because the transmission of this tradition is declining, but also because of the extinction of traditional mushrooms such as the ecomycorrhizal "loyo" (*Boletus loyo* Phil.) and "changles" (*Ramaria* spp.) among others. These species often fructify in fairy rings associated with trees of the genus *Nothofagus*, specifically the evergreen *N. dombeyi* and the deciduous *N. obliqua*. Both *B. loyo* and *Ramaria* spp. are important sources of food, but have become less frequent or have already disappeared entirely, especially in sites with strong anthropogenic intervention, e.g. in the form of logging [15]. Therefore, the Chilean Ministry of the Environment ~~has recently declared the "loyo" as~~recently declared the "loyo" an endangered species. In addition, it should be noted that Panguipulli is the commune with a notable rapprochement between collectors of wild edible mushrooms and researchers. This has led to several workshops for the exchange of knowledge, thus creating a record of wild mushrooms known by collectors in the territory [16].

This paper presents findings from a research project carried out in southern Chile (Fig 1A). In the framework of the project, the diversity of ECM fungi associated with *Nothofagus* spp. from fruiting bodies and ECM tips was studied. The aim was to develop methods of propagation of edible wild mushrooms and their inoculation of native forest plots in order to promote sustainable mushroom collection as an alternative for the diversification of family

Commented [xxx1]: The Figure 1A presents a map with a yellow square. Please, add information about the square.

farming [17]. As part of this project, we have ~~for several years been conducting research and collecting fruiting bodie~~been conducting research and collecting fruiting bodies for several years, specifically targeting edible ECM mushrooms from these forests.

Here, we provide information about ECM fungal communities by sampling mycorrhizal root tips from *N. dombeyi* and *N. obliqua* from both pure and mixed forests and addressing the following specific questions:

1. What is the diversity of fungi associated with roots of ~~*Nothofagus*~~ *N. dombeyi* and *N. obliqua* from pure and mixed forests?
2. What is the relative belowground abundance of the wild edible mushrooms *Boletus loyo*~~.~~ and *Ramaria* spp. in the sampled forests?
3. Do biotic and/or abiotic factors have an impact on the fungal communities in general and the edible mushrooms ~~*Boletus*~~ *B. loyo* and *Ramaria* spp. in particular?

We hypothesize that the ECM fungal communities differ across evergreen-deciduous *Nothofagus* forests represented either by pure or mixed stands of *N. dombeyi* and/or *N. obliqua*. This continuum can be correlated to changes in biotic and/or environmental abiotic variables such as ~~chemical properties of the soil~~soil chemical properties, plant species and tree age. We aim to contribute to the knowledge of the diversity and relative abundance of ECM fungal communities associated with the roots of these two *Nothofagus* species. A special focus thereby is to characterize the relative abundance of the edible fungi ~~*Boletus*~~ *B. loyo* and *Ramaria* spp. associated with roots from two different species of *Nothofagus*. The results generated here are intended to promote actions for these two sources of food and other species of mushrooms in order to apply appropriate sustainable silvicultural management, conservation decisions and restoration~~s~~ actions.

Material and methods

Study site

The study area is located at the northeast of the Los Ríos region, Chile, commonly known as the "seven lakes" due to the number of lakes it hosts, bordering the La Araucania region to the north and Argentina to the east. Specifically, most of the sites are located within the commune of Panguipulli, which is the largest commune of the region with an area of 3.292 km^2 (Fig 1A). Sites PR, PC and PP are located further north within La Araucania region, while sites RR, CC, MR, NC and MCR are within the Los Ríos region (Fig 1B). Geologically, the sites are part of the Andean foothills whose origin corresponds to the Quaternary period ~~characteriszed~~ characterized by intense volcanic activity and periods of glaciation that gave rise to various forms of morainic, fluvioglacial and glaciolacustrine deposits. The climate is temperate-rainy with mediterranean influences and the precipitation is concentrated in the winter months (between May and August) with ranges between 567 and 623 mm per month. The average temperature reaches 11.5°C with the warmest months fluctuating between 13°C and 16°C and the winter season months reaching temperatures in the order of 7°C [18].

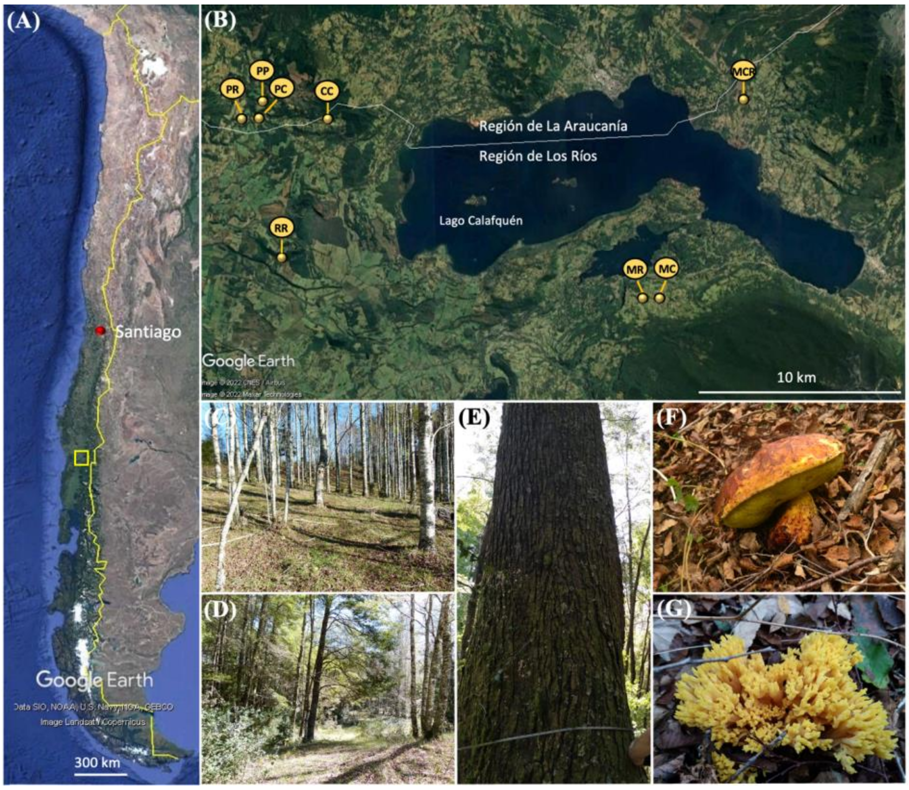

Fig 1. Geographic location and sampling sites and edible fruiting bodies in the communes of Loncoche of La Araucania and Panguipulli of Los Ríos regions (Chile).

(A) Map of Chile, indicating the geographic location where the material was collected (map data: Google Earth, ©2021). (B) Collection sites in the Loncoche commune: (PR) Pultro road, (PC) Pultro road and (PP) Pultro meadow. Collection sites in the Panguipulli commune: (RR) Caricuicui (CC), Linda Flor (MR), Pullinque Alto (MC) Pullinque Alto and (MCR) Pukura (map data: Google Earth, ©2021). (C) Young pure stand of *Nothofagus obliqua*. (D) Young mixed stand of ~~*Nothofagus*~~ *N. dombeyi* and *N. obliqua*. (E) Old tree of *N.~~othofagus~~ dombeyi*. (F) Mature fruiting body of *Boletus loyo*. (G) Fruiting body of *Ramaria* sp.

The forest in the study area is dominated by the evergreen ectomycorrhizal species *Nothofagus dombeyi* (Mirb.) Oerst. ("coihue") and the deciduous *N. obliqua* (Mirb.) Oerst. known as "hualle" in the young growth stage or "pellín" in the case of mature trees. The distribution patterns of these two species are rather linked to different climate and soil conditions; *N. dombeyi* is widespread and occurs best on humid slopes of the west side of the

Andes on relatively shallow soil, becoming a pioneer species in the Andes Cordillera (cite). *N. obliqua* is ~~in contrast a rather thermophilic species which grows on deep soil~~a rather thermophilic species that grows on deep soil, especially in the intermediate depression [19,20]. The forests PR/PC and MR/MC are mixed stands, forest CC with *N. dombeyi*, and forests PP, RR, and MCR with *N. obliqua* are pure stands. Fruiting of *B. loyo* and *Ramaria* ssp. occurred often in fairy rings from early April until the end of May. Fruiting bodies of *B. loyo* were common in sites PR, MR, MC, and PCR, whereas *Ramaria* ssp. were abundant in sites RR, CC, MCR, and PPR (Fig 1F-G). All these forests consist of > 300 years old individual trees surrounded by secondary growth of mostly < 70-year old trees [20]. S1 Table details the main plant species forming part of the understorey at each sampled forest site.

Commented [xxx2]: Please consider addressing a critical imbalance in the representation of forest stands, specifically the limited number of forest types. I would highly recommend including a discussion on the limitations of the study in the relevant section.

Commented [xxx3]: Could you please help me verify the accuracy of the term 'PPR'? I couldn't locate it on the map.

Root and soil sampling

During the summer (January) of 2021, root samples were collected from sites either from monospecific forests composed of *N. obliqua* or *N. dombeyi*, or where both species were mixed (Fig 1C-E). The collection sites PR and PC were neighbours to each other or even had some degree of overlap between them, the same applies for sites MR and MC. In these sites, *N. obliqua* and *N. dombeyi* co-occurred, but each species was sampled and ~~analys~~z~~ed~~ analyzed separately. At each site, 12 trees were randomly selected and root samples were obtained from each: 6 young trees (Y) and 6 mature trees (O) for which the diameter at breast height (DBH) was recorded, where DBH ≤ 7 cm was considered as a young tree and DBH ≥ 43 cm as mature trees, respectively. Root samples and soil covering these were collected within fairy rings at 0.2 m, 0.4 m and 0.6 m from the base of the trunk for young trees (Y), and 0.5 m, 1 m and 1.5 m for mature trees (O), at a depth of 20 cm following the direction of the ground slope downwards. Root sampling from both *Nothofagus* species was especially challenging because their roots grow deep into the soil and therefore, it was not possible to collect ECM tips beneath the fairy rings (beneath the fruiting bodies). A total of 96 samples containing root and soil were placed in plastic bags, labelled, and stored in a cooler for transport to the Mycology Laboratory belonging to the Universidad Austral de Chile for storage at 4°C until processing.

Root processing and morphotyping

The roots of *N. dombeyi* and *N. obliqua* were separated from the soil and washed with distilled water to remove soil particles adhering to their surface. From each sample, roots were deposited into Petri dishes for examination under a Zeiss stereomicroscope at 10x and 40x

magnification. ECM root tips were then classified primarily based on their morphology, i.e., branching type, size, colour, and presence of emanating hyphae and rhizomorphs [21]. ~~From those dominant morphotypes, two to five ECM root tips were chosen arbitrarily~~Two to five ECM root tips were chosen arbitrarily from those dominant morphotypes. From each sample, a total of 100 ECM tips were obtained and placed in a sterile 1.5-ml Eppendorf tube, and air dried at 50°C over~~-~~night. In total, 9,600 ECM root tips, half from young and mature plants each were thus sampled for *N. dombeyi* and *N. obliqua*. Additionally, a part of the root system from each sampled plant is preserved in 70% ethanol and refrigerated at 4°C.

DNA extraction and PCR amplification

Genomic DNA was extracted from 96 pooled ECM tip samples using the DNAeasy® PowerSoil® Pro kit (QIAGEN, Hilden, Germany), following the manufacturer's recommendations. First, previously dried roots were incubated for 30 min in the presence of buffer and macerated at least 3 times with a sterile pistil. Next, samples were ground to a fine powder by placing tissues in a 2.0-mL screw-cap tube containing a single 3.0-mm and five to ten 1.5-mm stainless steel beads and shaking them in a BeadBug™ microtube homogenizer (Sigma-Aldrich, Missouri, USA) for 120 seconds at a speed setting of 400 rpm. The extracted genomic DNA was stored at -20°C for later use.

To amplify the internal transcribed spacers (ITS region including the 5.8S), we used the primers ITS1F [22] and ITS4 [23]. PCR profiles and temperature programs were the same as in Schön et al. [24].

Library preparation and Illumina sequencing

The quality of amplified DNA was determined by electrophoresis using a 0.8% agarose gel and by DNA quantification using a 133 Nano Spectrophotometer (NanoDrop Technologies Inc., Wilmington, DE). For Illumina sequencing, PCR products were pooled 2 to 1 within each site and tree age, resulting in a total of 48 pooled PCR products for sequencing on the Illumina platform (Table S2). The primers ITS1FI2_A (5'-GAACCWGCGGGGARGGATCA-3') and ITS2_A (5'-GCTGCGTTCTTCATCGATGC-3') from the work of Schmidt et al. [25] were used to sequence both DNA strands. The DNA pool from the mixture was prepared for sequencing using the library denaturation and dilution guide, according to Fadrosh et al. [26]. Pairwise sequencing was performed using the MiSeq Reagent Kit v3 (2 × 300 cycles) on the

MiSeq Illumina sequencing platform at the AUSTRAL-omics Core-Facility (Faculty of Science, Universidad Austral de Chile; https://australomics.cl/).

Analysis for the identification of fungal communities

The demultiplexing process was performed from the raw sequences generated by the Illumina MiSeq with cutadapt 2.10 [27]. Briefly, cutadapt identifies and separates sequences from each index in R1, then identifies and groups sequences that match the index in R2. This allows the separation of each ~~of the samples~~sample, depending on the combination of indexes used.

A filtering process was carried out on sequences with a quality lower than Q30. For this, two programs, Trimomatic 0.39 [28] and PRINSEQ 0.20.4 [29], were used to remove sequences from Illumina sequencing and poor quality full or partial reads. Subsequently, the paired sequences (forward and reverse) were assembled using Pandaseq 2.11 [30]. Finally, all sequences with a length below 150 bp were removed.

We removed potentially chimeric sequences using the ‘uchime_ref’ option in vsearch 2.15.1 [31] and the same database that was used for taxonomic annotation. All non-chimeric sequences were then taxonomically identified by the MALT 0.5.3 pipeline [24,32], using the NCBI fungal ITS RefSeq database (PRJNA177353, retrieved 7. September 2021). MALT performs semiglobal alignment of reads to full-length reference sequences and assigns taxonomic labels based on the weighted lowest common ancestor algorithm applied to the top 5 percent of hits. The results from MALT were tabularized using tools provided by MEGAN 6.21.12 [33] and summarized using ete3 3.1.2 [34].

In order to compare the composition of the dominant elements of the fungal communities at the different sampling sites, the relative abundance of the most abundant fungal orders was visualized as stacked bar charts in seaborn 0.11.2 [35].

Since the ITS RefSeq project lacks a reference sequence for the common mushroom species *B.~~oletus~~ loyo*, we additionally compared reads against the available sequence KY462402.1, using the same approach as described above (MALT+MEGAN).

Ecological ~~characteriszation~~ characterization of fungal communities

In addition to taxonomic classification, the lifestyle of a fungal taxon and the interactions of different lifestyles in a community are important aspects of fungal ecology. Therefore, all taxa (on the level of genus) were classified into ecological guilds with the FUNGuild tool v1.2 and database (the database is available at www.stbates.org/funguild_db.php)[36]. The database currently consists of more than 13,000 records.

The alpha diversity of fungal communities was assessed by calculating Hill numbers [37] based on the normalized counts on the genus level in the R package vegan 2.6-4 [38]. As described in Schön et al.[24], Hill numbers are a general measure of biodiversity, defined as a reciprocal mean proportional abundance and differently weighing taxa based on their abundance, i.e. with different weighting of rare and abundant species or taxa. Hill numbers can be computed with different values for the order of diversity q, where values of $q<1$ favour rare taxa (and $q = 0$ simply corresponds to taxon richness), and $q>1$ give more weight to common species or taxa. They also encompass other common ~~indices of diversity~~diversity indices such as Shannon ($q = 1$ is equivalent to the exponential of the Shannon index), Simpson ($q = 2$ is equivalent to the inverse of the Simpson index), etc.

Finally, the relationship between genus composition of the samples (using only genera with at least 0.1% relative abundance) and the measured environmental parameters such as inclination, sodium content and clay portion of the soil was visualized by non-metric multidimensional scaling (NMDS) based on Bray-Curtis distances in the vegan package (k=5)[38]. Environmental variables were fitted using the ‘envfit’ function of vegan. Significance of the variables were assessed with 999 random permutations and only significant variables ($p<0.01$) were plotted.

Results

Illumina sequencing

For the pooled samples, the numbers of raw reads ranged from 21622 to 218311 and after filtering varied between 8813 and 156496 (S2 Table). The number of assembled reads ranged from 8101 to 103199 reads with a maximum length of 557 bp. The percentage of chimeric sequences ranged from 1.5% to 49.2% for assembled sequences with a minimum length of 150 bp.

Taxonomic coverage and diversity of fungal communities associated with root tips and surrounding soil from *Nothofagus dombeyi* and *N. obliqua* forests

In total, 1125 fungal taxa on the genus level were identified (S3 Table). These were distributed over 131 orders (S4 Table) representing the phyla Ascomycota (34.5% of total non-chimeric reads), Basidiomycota (62.1%) and Mucoromycota (3.1%), whereas Chytridiomycota, Cryptomycota, Olpidiomycota and Zoopagomycota where each represented by less than 1% of the total reads (Fig 2 and S5 Table).

At most sites and ~~at~~ both ages of the tree species sampled, taxa of the orders Agaricales, Helotiales, Thelephorales, Cantharellales, Trichosporonales, and Sebacinales were the most abundant (at least 4% mean relative abundance). At sites CC, MCR, and PPR both adult trees of *N. dombeyi* and *N. obliqua* showed a higher relative abundance of some orders such as Agaricales than younger trees (Fig 2). On the contrary, at sites MC, MR, and PR young trees of *N. dombeyi* and *N. obliqua* showed a higher relative abundance of Agaricales. For Helotiales, a high abundance was detected at sites CC, MC and MCR with old trees and sites MR, PPR and RR with young trees. Thelephorales were abundant at sites MC, MR, and PR with old trees and at sites CC, MCR, PC, PPR, and RR with young trees. For the case of Cantharellales, in all sites (except sites CC and RR), the young trees showed a higher abundance. Sebacinales exhibited higher abundance exclusively in old trees from sites CC and RR, while in all other

Commented [xxx4]: Could you please name the Sheet1 in the Excel file as "S3" or "fungal taxa"?

Commented [xxx5]: Similar to S3. Please consider naming the Sheet1.

Commented [xxx6]: Similar to S3 and S4. Please consider naming the Sheet1.

Commented [xxx7]: Could you please provide clarification regarding whether we are referring to plots or sites? It would help ensure a clear understanding of the context.

Commented [xxx8]: Check.

Commented [xxx9]: Could you please review the PPR plot or site and determine whether the x-axis should represent 'plot' or 'site'? Additionally, please make the necessary adjustment in the Figure 2.

sites, they were predominantly found in young trees. ~~Sebacinales were more abundant only in old trees from sites CC and RR, whereas in all remaining sites were more dominant in young trees~~.

At the genus level, the presence of *Boletus* (*B. loyo*) and *Ramaria* (*Ramaria* spp.) was nil or very low in relative abundance from those sites where fruiting bodies of these genera were observed (S3 Table).

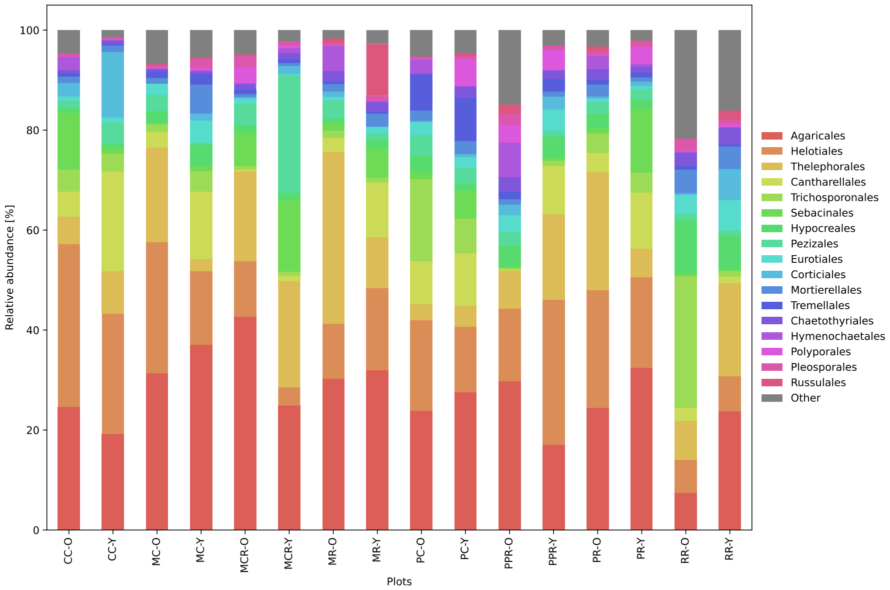

Fig 2. Relative abundances of the fungal communities at the order level detected in root tips and surrounding soil from *Nothofagus dombeyi* and *N. obliqua* forests in mature (O) and young (Y) trees. For site abbreviations, see Fig 1. Fungal orders are ordered according to their mean relative abundances. Orders with a mean relative abundance of <1% are grouped into 'Other'.

Commented [xxx10]: Could you please review whether it would be preferable to use 'plot' or 'sites'? Additionally, please verify the correctness of 'PPR'.

Commented [xxx11]: Stands?

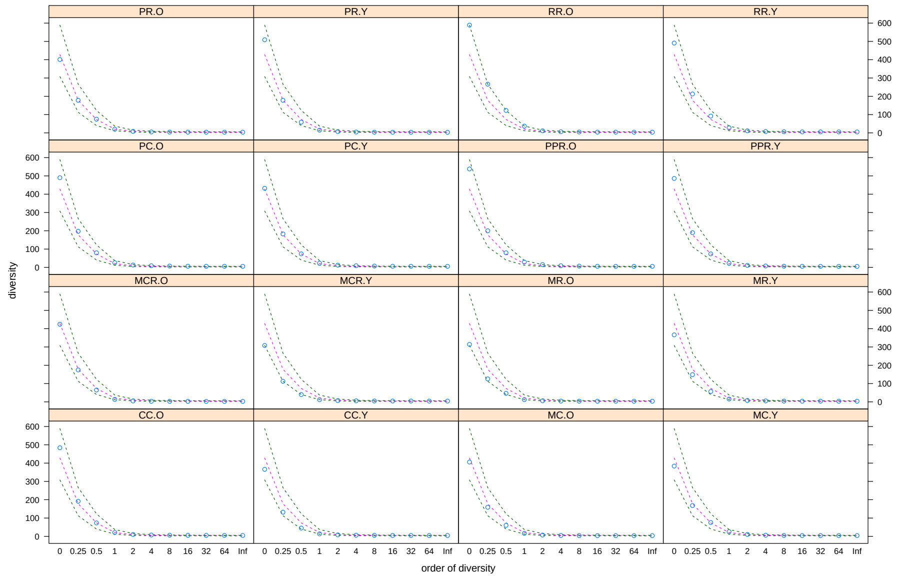

Fig 3. Alpha-diversity (circles) of belowground fungal communities associated with root tips (ECMs) and surrounding soil from specific and mixed evergreen-deciduous *Nothofagus* ~~*bombeyi*~~ *dombeyi* and *N. obliqua* forests.

For site abbreviations, see Fig. 1. Each sample is labelled according to the sampling site and the age of the sampled tree (O = old and Y = young). For each sample, Hill numbers for different ~~values of qq~~ values (order of diversity) were calculated, giving different weights to species abundances (and q=0 corresponding to species richness). Green dotted lines indicate minimum and maximum across all samples, and pink dotted lines correspond to the median.

The diversity indices of the belowground fungal communities associated with ~~*Nothofagus*~~ *N. dombeyi* and *N. obliqua* forests ~~are~~ were illustrated in Fig 3 (S6 Table). The highest taxon diversity (with q=0, corresponding to species richness) was found in mature trees at site RR (589 genera detected), whereas the lowest diversity was found in young trees at plot MCR (308 genera). ~~In general, we~~We generally observed a slight trend of lower diversity observed in young trees vs. mature trees (easily observable, e.g. at sites RR, MCR, and CC).

Comparing fungal communities among sampling sites

The composition of overall fungal communities assessed by two-dimensional NMDS plot based on Bray-Curtis dissimilarities did not show marked differences among the

Commented [xxx12]: Please capitalize the first letter of the axis titles. For example, 'diversity' should be changed to 'Diversity,' and 'order of diversity' should be changed to 'Order of diversity.

Commented [xxx13]: Ectomycorrhizae?

Commented [xxx14]: Stands?

Commented [xxx15]: Please provide the recommended dimensional solution for the NMDS, including the final stress and final instability values.o

stands~~forests~~ sampled (Fig 4). Moreover, the fungal community composition was not significantly different between young and old trees. Similarly, the soil properties and forest structure (monospecific vs. mixed forests) did not seem to have a significant influence on fungal community composition, although non-significant trends could be observed, e.g. along the gradient of total N concentration in the forest soils. The sites could also not be distinguished based on their host trees, but a trend could be observed here as well with some sites overlapping more than others, importantly the sites from mixed stands MR/MC and PR/PC.

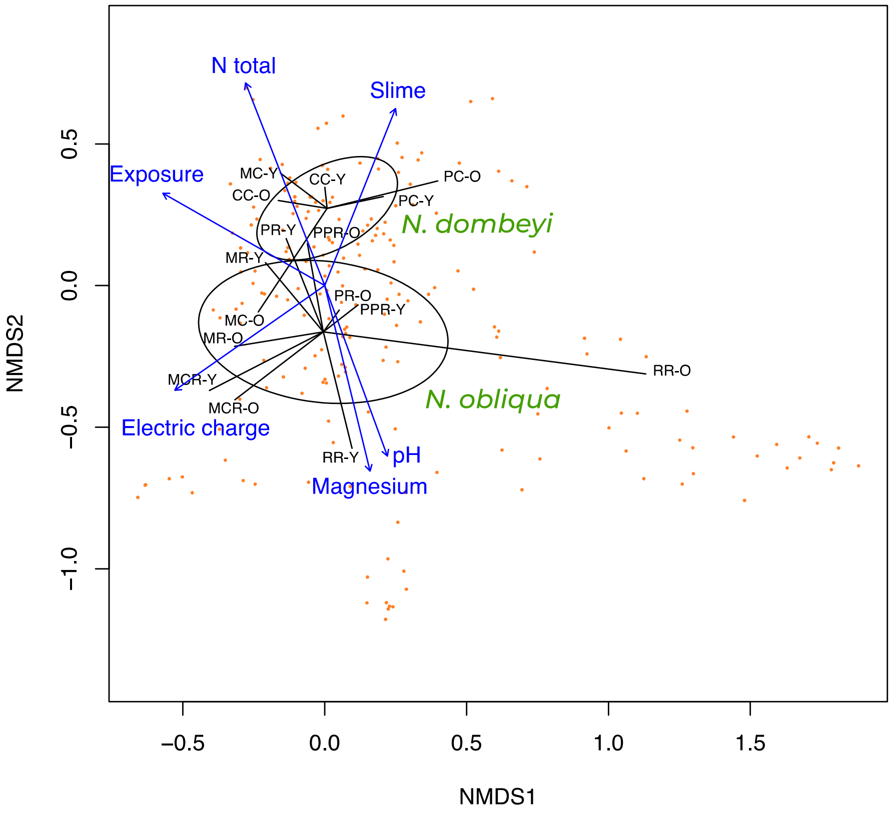

Fig 4. NMDS plot of fungal communities associated with root tips (ECMs) and surrounding soil from eight monospecific and mixed *Nothofagus* ~~b~~d*ombeyi* and *N. obliqua* ~~forests~~ stands based on ITS2 sequences.

For site abbreviations, see Fig 1. ~~Individual fungal genera with a minimum relative abundance of 0.1% are represented by red dots~~Red dots represent individual fungal genera with a minimum relative abundance of 0.1%.

Commented [xxx16]: It would be helpful to rename 'NMDS1' as 'Axis 1' and 'NMDS2' as 'Axis 2' for improved clarity in the Figure 4.

Commented [xxx17]: Please indicate where the "Exposure" variable was come from. Do it in the methods sections please.

Environmental variables with p<0.01 are displayed as blue arrows. Samples are grouped according to the corresponding tree species and marked as coming either from mature (O) or young (Y) trees.

Ecological diversity of fungal communities associated with root tips (ECMs) and surrounding soil of young and ~~mature~~ old trees of *Nothofagus* ~~bd~~*dombeyi* and *N. obliqua*

Formatted: Font: Bold

The fungal taxa were grouped into 7 broad ecological categories; the most common associations were saprotroph with a median of 124 taxa, pathotroph with 52 taxa, pathotroph-saprotroph-symbiotroph, pathotroph-saprotroph, and symbiotroph with a median between 33 and 43 taxa each (Fig 5, S3 Table). ~~No major differences in relation to~~There are no major significant differences in the number of taxa in each category between young and old *N. dombeyi* and *N. obliqua* trees ~~was~~ were found.

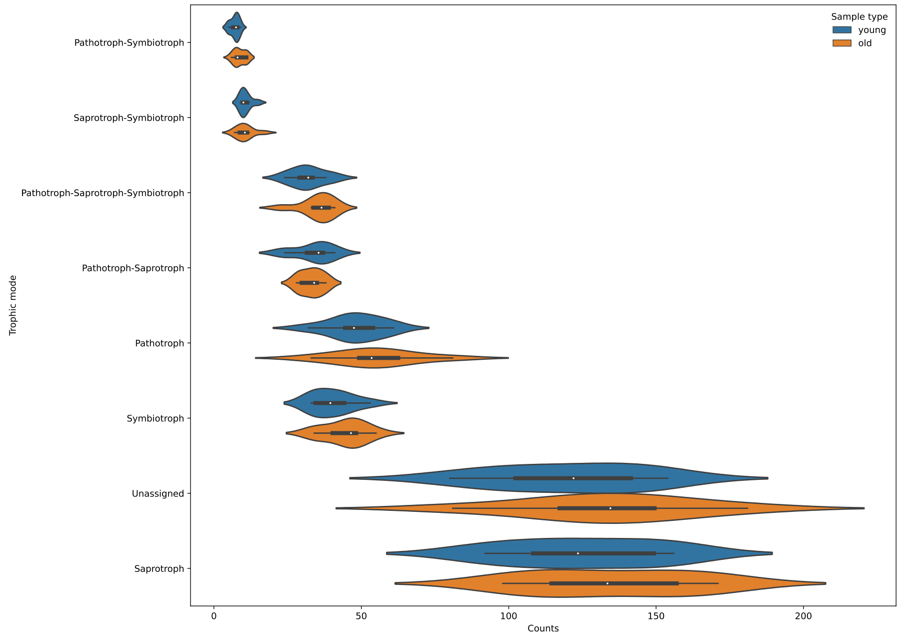

Fig 5. Ecological associations of taxa from fungal communities in root tips and surrounding soil from *Nothofagus dombeyi* and *N. obliqua* forests. Samples from ~~mature (~~old~~)~~ and young trees are compared. Broad ecological categories were predicted with FunGuild on the level of genus. ~~The median values in each group are represented by a white dot~~A white dot represents the median values in each group, the interquartile range corresponds to the black bar in the center of each violin. The kernel density plot for each category is displayed in blue and orange, corresponding to the tree age.

Commented [xxx18]: Unfortunately, I was unable to find the figure in a separate file, which made it difficult for me to view it properly. Could you please ensure that all the relevant files, including the figure, are attached so that I can have a complete understanding?

Discussion

Commented [xxx19]: Could you please provide a discussion on formulating hypotheses?

Diversity of fungal communities in *Nothofagus dombeyi* and *N. obliqua* forests

Formatted: Font: Italic

Our results reveal a high diversity of fungi associated with *N. dombeyi* and N. *obliqua* (see Figs 2 and 3). In general, the fungal groups detected here are in agreement with previous studies conducted in other South American *Nothofagus* forests [3,4] and even from studies conducted in boreal and temperate forests from the N~~n~~orthern H~~h~~emisphere [e.g. 24]. Although only ECM tips were sampled, the approach allowed us to sequence other fungal groups with functions ranging from saprotrophic to pathogenic (see Fig 5).

Similar to the study by Nouhra et al. [3], we observed no statistical differences in the main fungal partners associated with *N. dombeyi* and *N. obliqua*. ~~Perhaps t~~This result ~~reflects the fact~~ suggest that both plant species practically coexist at the sampled sites and remaining mature trees after logging served as sources of fungal inoculum for the new generation of trees [39]. Additionally, both ~~plant~~ tree species grow on soil with broadly similar characteristics~~,~~ ; they have similar ages and are phylogenetically related. Although some sites showed greater anthropogenic intervention of both forest and soil use, those do not seem to ~~have a significant impact on~~significantly impact the diversity of the belowground fungal communities (see Fig 4).

Discrepancies between fruiting body occurrences and ectomycorrhizal root tip sequencing: the cases of *Boletus loyo* and *Ramaria* spp.

Our results reveal a high diversity of fungi compared to the limited number of fungal species represented by fruiting bodies that can be observed in the field. Several previous studies, both in the ~~northern~~ Northern ~~hemisphere~~ Hemisphere a~~s well as~~nd in South America, indicate that fungal diversity studies based on DNA sequencing allow the detection of fungal groups without conspicuous fruiting bodies. For example, Atheliales, Thelephorales,

Sebacinales (Basidiomycota), Helotiales, and Pezizales (both Ascomycota) either do not produce any fruiting bodies or develop inconspicuous fruiting bodies and ~~are~~, therefore often not detected in field sampling. It should be noted that the fungal groups detected as dominant in our study are the same as those that dominate in boreal and temperate forests in the northern hemisphere [e.g. 24]. ~~In our barcoding analysis,~~Our barcoding analysis shows the absence or low relative abundance of ~~*Boletus*~~ *B.* *loyo* and *Ramaria* spp. at sites where they are abundant in terms of fruiting bodies could be due to sampling bias, specifically because roots were taken very close to the trunk and at shallow depths. In contrast, both species grow in circles (fairy rings) around the trees, which could not be sampled mainly due to deep root growth below the fruiting bodies. Although Lian et al. [40] found that the ECM communities within and outside of the fairy rings were similar, there are also other cases where it has been observed that the aboveground and belowground occurrences of ECM species do not overlap [e.g. 41].

Impact of using metabarcoding for sustainable forest management decisions

Despite the enormous efforts in monitoring ecosystems with traditional methods based on visual identification and cultivation, these provide an incomplete assessment of the diversity that exists within them [42,43]. This issue is a major impediment in ~~the study of microorganisms such as fungi, which do not all form fructifications, are practically invisible to the human eye, or their fructifications~~ studying microorganisms such as fungi, which do not all form fructifications, are practically invisible to the human eye, or are so ephemeral that they easily escape field sampling. In addition, ~~a large majority of~~most fungi are not cultivable under laboratory conditions as they establish obligate parasitic or symbiotic associations with plants. Consequently, monitoring ~~of biodiversity and ecosystem functionality based on DNA sequencing offers advantages over traditional methods as it allows a more complete diversity to be surveyed and allows fungi to be detected from their vegetative structure (hyphae or mycelium), regardless of whether they~~ biodiversity and ecosystem functionality based on DNA sequencing offers advantages over traditional methods as it allows a complete diversity to be surveyed and allows fungi to be detected from their vegetative structure (hyphae or mycelium), regardless of whether they are fruit or not. It should be noted that the production of fruiting bodies involves various environmental and biological factors specific to each species, ~~which means that~~meaning some fungi may fructify very sporadically, including *B. loyo* and *Ramaria* ssp. [44]. More

accurate taxonomic identification (DNA barcoding) of fungal species can also be achieved ~~and~~, thus more detailed tracking of particular species. In conclusion, metabarcoding is a useful tool for detecting changes in particular taxa or the entire biodiversity, opening the possibility of being able to assess the ecological status of a given ecosystem according to such metrics. Potential benefits of metabarcoding of fungal communities for fungal monitoring programmes include ~~characteriszation~~ characterization of their food webs, assessment of response to disruption and stress, detection of sensitivity, rare, invasive, edible, toxic species, etc.

To conclude, our study represents a first glance at the fungal communities hosted by young and old *N. obliqua* and *N. dombeyi* trees ~~by using environmental ITS2 sequencing in an area considered as~~using environmental ITS2 sequencing in an area considered a biodiversity hotspot of southern Chile. Besides the characterization of ECM fungi, our results provide a view of a greater diversity of fungi that include previously undetected species (i.e. not detected as fruiting bodies). These findings reveal a high diversity and non-significant compositional differences of belowground fungal communities among *N. obliqua* and *N. dombeyi* trees, suggesting an impact of recolonization, similar anthropogenic use and soil conditions ~~as well as~~, and proximity between sites sampled.

Our study further highlights an attempt to link below- and aboveground occurrences of the edible mushrooms *B. loyo* and *Ramaria* ssp. As we detected great variations in the abundance of fruiting bodies vs. the abundance in the rhizosphere, these differences must be considered in the future to fully understand the distribution of these taxa; With this study, we hope to offer a starting point for future research in this area.

Commented [xxx20]: Could you please provide a concise paragraph discussing the potentialities of metabarcoding and the implications for your study for the rehabilitation, restoration, and, if possible, explicit forest management (e.g. shelterwood cutting)?

Acknowledgements

We thank C. Stuardo, I. Montenegro, V. Claramunt and E. Molina for carried out the collection of material and by providing pictures of collecting sites and fruiting bodies. We thank all the researchers involved in the project FYT-2018-0723. Finally, we thank J. Palma and E. Molina for inviting us to be part of and contribute to the project. MES would like to thank M.G. Fischer for their support.

References

1. Deckmyn G, Meyer A, Smits MM, Ekblad A, Grebenc T, Komarov A, et al. Simulating ectomycorrhizal fungi and their role in carbon and nitrogen cycling in forest ecosystems. Can J For Res. 2014;44: 535–553. doi:10.1139/cjfr-2013-0496

2. Barroetaveña C, Salomón MES, Bassani V. Rescuing the ectomycorrhizal biodiversity associated with South American Nothofagaceae forest, from the 19th century naturalists up to molecular biogeography. Forestry: An International Journal of Forest Research. 2019;92: 500–511. doi:10.1093/forestry/cpz047

3. Nouhra E, Urcelay C, Longo S, Tedersoo L. Ectomycorrhizal fungal communities associated to Nothofagus species in Northern Patagonia. Mycorrhiza. 2013;23: 487–496. doi:10.1007/s00572-013-0490-2

4. Truong C, Gabbarini LA, Corrales A, Mujic AB, Escobar JM, Moretto A, et al. Ectomycorrhizal fungi and soil enzymes exhibit contrasting patterns along elevation gradients in southern Patagonia. New Phytol. 2019;222: 1936–1950. doi:10.1111/nph.15714

5. Almonacid-Muñoz L, Herrera H, Fuentes-Ramírez A, Vargas-Gaete R, Larama G, Jara R, et al. Tree Cover Species Modify the Diversity of Rhizosphere-Associated Microorganisms in Nothofagus obliqua (Mirb.) Oerst Temperate Forests in South-Central Chile. Forests. 2022;13: 756. doi:10.3390/f13050756

6. INFOR. Productos forestales no madereros. 2019 pp. 1–26. Report No.: 33.

7. Cano-Estrada A, Romero-Bautista L. Economic, nutritional and medicinal value of edible wild mushrooms. Revista chilena de nutrición. 2016;43: 75–80. doi:10.4067/S0717-75182016000100011

8. Rugolo M, Mascoloti Spréa R, Dias MI, Pires TCSP, Añibarro-Ortega M, Barroetaveña C, et al. Nutritional Composition and Bioactive Properties of Wild Edible Mushrooms from Native Nothofagus Patagonian Forests. Foods. 2022;11: 3516. doi:10.3390/foods11213516

9. Jacinto-Azevedo B, Valderrama N, Henríquez K, Aranda M, Aqueveque P. Nutritional value and biological properties of Chilean wild and commercial edible mushrooms. Food Chemistry. 2021;356: 129651. doi:10.1016/j.foodchem.2021.129651

10. INFOR. Anuario forestal 2017. 2017 pp. 1–188. Report No.: 159.

11. Pino A, Cardyn P, Panguipulli G de T. La reserva de la biosfera de los bosques templados lluviosos de los andes australes y las singularidades territoriales de la comuna de Panguipulli. In: Moreira-Muñoz A, Borsdorf A, editors. Reservas de la Biosfera de Chile: Laboratorios para la Sustentabilidad. Santiago: Academia de Ciencias Austriaca, Pontificia Universidad Católica de Chile, Instituto de Geografía; 2014. pp. 190–207.

12. CONAF. Catastro y Actualización, Recursos Vegetacionales de Chile. Región De Los Ríos: Corporación Nacional Forestal; 2014.

13. INE. Censo 2017: Primera entrega de resultados definitivos - Cantidad de personas por sexo y edad. 2017.

14. Molares S, Toledo CV, Stecher G, Barroetaveña C. Traditional mycological knowledge and processes of change in Mapuche communities from Patagonia, Argentina: A study on wild edible fungi in Nothofagaceae forests. Mycologia. 2020;112: 9–23. doi:10.1080/00275514.2019.1680219

15. Barrena Ruiz J, Hernando Arrese M, Rojas Marchini F. Historical background of the Panguipulli Forestry and Timber Complex, Valdivia province, South-central Chile. Bosque (Valdivia). 2016;37: 473–484. doi:10.4067/S0717-92002016000300004

16. Palma Martínez J, Claramunt V, Montenegro I, Molina E. Protocolo experiencia de monitoreo de hongos silvestres comestibles. Santiago de Chile: FAO y MINAGRI; 2021 p. 36. doi:10.4060/cb0842es

17. Palma Martínez J, Claramunt V, Molina Rademacher E, Montenegro I, Chung Guin-po P. Manual para la recolección y manejo sustentable de hongos silvestres comestibles. El caso de loyo, changle, gargal y diweñe. INFOR; 2021. doi:10.52904/20.500.12220/31353

18. PLADECO. Actualización plan de desarrollo comunal de Panguipulli 2013-2017. Santiago, Chile; 2014.

19. Weinberger P. Beziehungen zwischen mikroklimatischen Faktoren und natürlicher Verjüngung araukano-patagonischer Nothofagus-Arten. Flora. 1973;162: 157–179. doi:10.1016/S0367-2530(17)31701-2

20. Veblen TT, Donoso C, Kitzberger T, Rebertus AJ. Ecology of Southern Chilean and Argentinean Nothofagus forests. In: Veblen TT, Hill RS, Read J, editors. The Ecology and Biogeography of Nothofagus Forests. New Haven, USA: Yale University Press; 1996. pp. 293–353.

21. Agerer R, editor. Colour atlas of ectomycorrhizae: with glossary. 1th-5th del ed. Schwäbisch Gmünd: Einhorn-Verl. Dietenberger; 1987.

22. Gardes M, Bruns TD. ITS primers with enhanced specificity for basidiomycetes - application to the identification of mycorrhizae and rusts. Molecular Ecology. 1993;2: 113–118. doi:10.1111/j.1365-294X.1993.tb00005.x

23. White TJ, Bruns T, Lee S, Taylor J. Amplification and direct sequencing of fungal ribosomal RNA genes for phylogenetics. In: Innis MA, Gelfand DH, Snisky JJ, White TJ, editors. PCR Protocols: A Guide to Methods and Applications. San Diego: Academic Press; 1990. pp. 315–322. doi:10.1016/B978-0-12-372180-8.50042-1

24. Schön ME, Nieselt K, Garnica S. Belowground fungal community diversity and composition associated with Norway spruce along an altitudinal gradient. PLOS ONE. 2018;13: e0208493. doi:10.1371/journal.pone.0208493

25. Schmidt P, Bálint M, Greshake B, Bandow C, Römbke J, Schmitt I. Illumina metabarcoding of a soil fungal community. Soil Biology and Biochemistry. 2013;65: 128–132. doi:10.1016/j.soilbio.2013.05.014

26. Fadrosh DW, Ma B, Gajer P, Sengamalay N, Ott S, Brotman RM, et al. An improved dual-indexing approach for multiplexed 16S rRNA gene sequencing on the Illumina MiSeq platform. Microbiome. 2014;2: 6. doi:10.1186/2049-2618-2-6

27. Martin M. Cutadapt removes adapter sequences from high-throughput sequencing reads. EMBnet.journal. 2011;17: 10–12. doi:10.14806/ej.17.1.200

28. Bolger AM, Lohse M, Usadel B. Trimmomatic: A flexible trimmer for Illumina sequence data. Bioinformatics. 2014;30: 2114–2120. doi:10.1093/bioinformatics/btu170

29. Schmieder R, Edwards R. Quality control and preprocessing of metagenomic datasets. Bioinformatics. 2011;27: 863–864. doi:10.1093/bioinformatics/btr026

30. Masella AP, Bartram AK, Truszkowski JM, Brown DG, Neufeld JD. PANDAseq: paired-end assembler for illumina sequences. BMC Bioinformatics. 2012;13: 31. doi:10.1186/1471-2105-13-31

31. Rognes T, Flouri T, Nichols B, Quince C, Mahé F. VSEARCH: a versatile open source tool for metagenomics. PeerJ. 2016;4: e2584. doi:10.7717/peerj.2584

32. Herbig A, Maixner F, Bos KI, Zink A, Krause J, Huson DH. MALT: Fast alignment and analysis of metagenomic DNA sequence data applied to the Tyrolean Iceman. bioRxiv. 2016;10.1101: 050559. doi:10.1101/050559

33. Huson DH, Beier S, Flade I, Górska A, El-Hadidi M, Mitra S, et al. MEGAN Community Edition - Interactive Exploration and Analysis of Large-Scale Microbiome Sequencing Data. PLOS Computational Biology. 2016;12: e1004957. doi:10.1371/journal.pcbi.1004957

34. Huerta-Cepas J, Serra F, Bork P. ETE 3: Reconstruction, Analysis, and Visualization of Phylogenomic Data. Molecular Biology and Evolution. 2016;33: 1635–1638. doi:10.1093/molbev/msw046

35. Waskom ML. seaborn: statistical data visualization. Journal of Open Source Software. 2021;6: 3021. doi:10.21105/joss.03021

36. Nguyen NH, Song Z, Bates ST, Branco S, Tedersoo L, Menke J, et al. FUNGuild: An open annotation tool for parsing fungal community datasets by ecological guild. Fungal Ecology. 2016;20: 241–248. doi:10.1016/j.funeco.2015.06.006

37. Hill MO. Diversity and evenness: A unifying notation and its consequence. Ecology. 1973;54: 427–432.

38. Oksanen J, Simpson GL, Blanchet FG, Kindt R, Legendre P, Minchin PR, et al. vegan: Community Ecology Package. 2022. Available: https://cran.r-project.org/web/packages/vegan/index.html

39. Cline ET, Ammirati JF, Edmonds RL. Does proximity to mature trees influence ectomycorrhizal fungus communities of Douglas-fir seedlings? New Phytologist. 2005;166: 993–1009. doi:10.1111/j.1469-8137.2005.01387.x

40. Lian C, Narimatsu M, Nara K, Hogetsu T. Tricholoma matsutake in a natural Pinus densiflora forest: correspondence between above- and below-ground genets, association with multiple host trees and alteration of existing ectomycorrhizal communities. New Phytol. 2006;171: 825–836. doi:10.1111/j.1469-8137.2006.01801.x

41. Gardes M, Bruns TD. Community structure of ectomycorrhizal fungi in a Pinus muricata forest: above- and below-ground views. Can J Bot. 1996;74: 1572–1583. doi:10.1139/b96-190

42. Goodwin KD, Thompson LR, Duarte B, Kahlke T, Thompson AR, Marques JC, et al. DNA Sequencing as a Tool to Monitor Marine Ecological Status. Frontiers in Marine Science. 2017;4. Available: https://www.frontiersin.org/articles/10.3389/fmars.2017.00107

43. Piombo E, Abdelfattah A, Droby S, Wisniewski M, Spadaro D, Schena L. Metagenomics Approaches for the Detection and Surveillance of Emerging and Recurrent Plant Pathogens. Microorganisms. 2021;9: 188. doi:10.3390/microorganisms9010188

44. Gonzalez, R. Estimación de la distribución potencial actual de las especies de hongos silvestres comestibles (Butyriboletus loyo, Ramaria sp., Cyttaria espinosae y Grifola gargal) en la región de Los Ríos, con énfasis en la comuna de Panguipulli. Tesis de Magister, Universidad de Chile. 2020.

Supporting information

S1 Table. Samples of the belowground fungal communities associated with *Nothofagus dombeyi* and *N. obliqua* forests.

S2 Table. Results of Illumina sequencing of ECM tips from pooled samples. The table shows the number of raw reads, assembled reads, filter reads longer than 150 bp and the number of reads after chimera checking.

S3 Table. Relative abundance of fungal communities at genus level from old and young *Nothofagus* forests. Trophic modes at genus level are given and those are used in Fig 6.

S4 Table. Relative abundance of fungal communities at order level from old and young *Nothofagus* forests. The mean values are provided an those are used in Fig 2.

S5 Table. Relative abundance of fungal communities at phylum level from old and young *Nothofagus* forests. The mean values are provided.

S6 Table. The diversity indices of the belowground fungal communities associated with *Nothofagus dombeyi* and *N. obliqua* forests. Diversity indices are visualized in Fig 3.
